# Wide Cost Variations Observed in Antibiotics and Analgesics Prescribed for Dental Care in India: A Price and Affordability Analysis

**DOI:** 10.7759/cureus.21755

**Published:** 2022-01-31

**Authors:** Jeevitha Gauthaman, Mathaiyan Jayanthi

**Affiliations:** 1 Pharmacology, Jawaharlal Institute of Postgraduate Medical Education and Research, Puducherry, IND

**Keywords:** out-of-pocket expenditure, health action international, dental diseases, analgesics, antibiotics

## Abstract

Introduction

With high prevalence rates across the population, dental pain is the most common symptom that drives a patient to visit the dentist. Dentists prescribe analgesics and antibiotic prophylaxis apart from routine dental treatments. The increasing costs of medications are a major factor that adds to the financial burden of patients seeking oral care.

Methods

A list of antibiotics and analgesics recommended by the American Dental Association for dental pain, swelling, and post-procedural pain management was analyzed for the cost variation and cost ratio. The affordability was calculated by the World Health Organization/Health Action International (WHO-HAI) methodology. The most expensive and the least expensive brands of all drug regimens were compared.

Results

Wide cost variations were observed between the different brands of antibiotics and analgesics. The highest cost variation was found in ampicillin 500 mg injection (13579.2%). In the affordability analysis; when the most expensive brands were analyzed, the lowest-paid unskilled worker had to lose 2.1 days of wages to afford the drug regimen. The price range was widest for injection ampicillin 500 mg (Rs. 4.24-580.0) and lowest for tablet ibuprofen 400 mg (Rs. 0.7-1.6).

Conclusion

Dental surgeons need to be well aware of the pharmacoeconomics behind medications that directly decides the affordability of treatments. Careful selection of drugs by the dentists considering their quality, availability, and costs is of pivotal importance. Prescribing generic medications and increasing the availability of drugs in public health sectors can help provide cost-effective dental care for all.

## Introduction

Dental conditions are one of the leading causes of disability that affect more than half of the world’s population at a given time [1]. In India, the prevalence of untreated dental caries in children below six years is estimated to be 49.6%, reflecting a child population of approximately 12 crores [2]. Dental pain is the most common symptom that drives patients to seek dental treatment [3]. In India, the prevalence of dental pain is 71% in 12-year-old children [4].

Dentists commonly prescribe analgesics like ibuprofen and acetaminophen and antibiotics like penicillin, cephalosporins, and fluoroquinolones as part of prophylaxis before dental procedures. These drugs are prescribed based on clinical guidelines laid down by professional bodies across the world. The American Dental Association (ADA) is one such pivotal organization that lays standard guidelines and recommendations for providing quality dental care. The ADA has recommended drug regimens for dental pain and swelling, which are classified into three categories [5]. During or after the course of medications, the dentist usually plans to alleviate the source of infection through procedures like scaling, root planning, root canal treatment, tooth extractions, flap surgeries, etc. However, lack of affordability forces a significant number of patients from lower-middle-income countries to avoid completing the course of medications and subsequently miss appointments. Apart from the cost of dental procedures, the increasing cost of medicines is a pressing concern that adds to the out-of-pocket expenditure of patients. The lack of essential medicines in public health facilities is also one of the important factors that need to be considered [6]. With around 3,000 pharmaceutical companies producing over 60,000 generic drugs, wide cost variations from 7.34% to 1049.82% are present among different brands of antibiotics in the Indian market [7].

In this article, we wanted to find the cost variation in antibiotics and analgesics prescribed for dental pain, swelling, and post-procedural pain management according to the ADA recommendations. The objectives of this paper are to find the cost variation in different brands of antibiotics and analgesics in the Indian market that is prescribed by dentists according to the ADA recommendations for dental pain and post-procedural pain management and to analyze the affordability of drugs prescribed for dental pain and swelling according to the World Health Organization/Health Action International (WHO-HAI) methodology.

## Materials and methods

A list of antibiotics given by the American Heart Association (AHA) for the prevention of infective endocarditis (2007) and recommended by the ADA for prescribing in dental procedures was obtained from the official website of ADA. The ADA has recommended exclusive drug regimens for dental pain and swelling, which are classified into three categories: categories A-C. Category A is comprised of drugs prescribed for pain and swelling in patients not allergic to penicillin. Category B is comprised of drugs recommended for patients (with pain and swelling) with an allergy to penicillin without any history of anaphylaxis, angioedema, or hives. Category C is comprised of drugs recommended for patients with pain and swelling and a history of anaphylaxis, angioedema, or hives with penicillin, ampicillin, or amoxicillin. These drug regimens along with their categories were obtained from an ADA report [5]. A set of analgesics recommended by ADA under “Oral Analgesics for Acute Dental Pain” (2020) was also obtained.

The prices of all the above drugs in INR were taken from the Current Index of Medical Specialties, 43rd edition, published in 2021. The cost/defined daily dose (DDD) was calculated by multiplying the unit cost of a particular strength of the formulation with the number of units required to maintain the DDD. All drugs included in the study were taken as a standard dose. The cost variation percentage was analyzed by finding the prices of the most expensive and the least expensive brand of every antibiotic/analgesic.

The percentage of cost variation was calculated as $\frac{Maximum\ cost - Minimum\ cost}{Minimum\ cost}$

The cost ratio was calculated as $\frac{Highest\ price}{Lowest\ price}$

For the affordability analysis, all the drug regimens recommended for pain and swelling classified under categories A-C were included. The affordability was measured using the WHO/HAI methodology, which states that a drug is considered unaffordable if a lowest-paid unskilled government employee loses more than a single day’s wages to pay for the drug [8]. In India, the Ministry of Labour and Employment (2021) lists the wages entitled for workers of different categories. The average wages for the lowest-paid unskilled worker were calculated as Rs. 375 per day [9]. The total wages (in days) that a worker has to lose to purchase a complete course of medicines from each of the categories were calculated separately. The medicines under each category were classified into two groups, namely the group with the most expensive brands and the group with the least expensive brands. These two groups were compared to find the cost variations. Since all the data used for this study was freely available in public domains and since no data related to humans or biological material were used, the study gained exemption from the Institutional Ethics Committee.

## Results

A total of 12 unique drugs comprising of 11 single drugs and one fixed drug combination were included in the analysis. There were two analgesics and 10 antibiotics, out of which eight antibiotics were recommended under the antibiotic prophylaxis for adults and seven antibiotics were recommended for children. Nine drugs were oral formulations, three were intramuscular injections, and three drugs were included as oral and intramuscular formulations.

Tablet amoxicillin with clavulanic acid (500 + 125 mg) had the highest number of brands (104) followed by tablet azithromycin 500 mg with 92 brands and tablet azithromycin 250 mg with 74 brands. Wide cost variation was seen among various brands of drugs. Injection ampicillin 500 mg had the highest cost variation (13579.2%) followed by tablet metronidazole 400 mg (2383.3%). The least-cost variation was seen in injection clarithromycin 500 mg (11.7%) followed by injection clindamycin 600 mg (16%). The maximum cost/DDD was seen in injection ampicillin 500 mg (3480.0) followed by injection clarithromycin 500 mg (2298.0). Tablet metronidazole 400 mg had the least cost/DDD (0.6) followed by tablet ibuprofen 400 mg (2.1) and tablet acetaminophen 500 mg (5.4). The cost ratio was highest for injection ampicillin 500 mg (136.8) and lowest for three drugs, namely injection clindamycin 600 mg (1.2), syrup azithromycin (1.2), and injection clarithromycin 500 mg (1.2). The price range was widest for injection ampicillin 500 mg (Rs. 4.24-580.0) and lowest for tablet ibuprofen 400 mg (Rs. 0.7-1.6) (Tables 1, 2).

**Table 1:** Cost ratio and cost variations of analgesics prescribed for dental pain and antibiotics given as prophylaxis for dental procedures ATC, Anatomic Therapeutic Chemical classification; DDD, defined daily dose; INR, Indian National Rupee.

S. No.	Items	Strength (mg)	Formulation	ATC code	DDD (mg)	No. of brands	Highest price (INR)	Maximum cost/DDD (INR/mg)	Lowest price (INR)	Minimum cost/DDD (INR/mg)	Price range (INR)	Cost ratio	% Cost variation
Analgesics													
1.	Ibuprufen	400	Tab.	M01AE01	1200	05	1.6	4.8	0.7	2.1	0.7-1.6	2.3	128.6
2.	Acetaminophen	500	Tab.	N02BE01	3000	23	3.3	19.8	0.9	5.4	0.9-3.3	3.7	266.7
3.	Acetaminophen	250	Tab.	N02BE01	3000	35	3.4	17.0	1.4	7.0	1.4-3.4	2.4	142.9
Drugs for antibiotic prophylaxis (Adults)													
1.	Amoxicillin	500	Inj.	J01CA04	1500	33	14.4	43.2	2.9	8.7	2.9-14.4	2.9	189.7
2.	Ampicillin	500	Inj.	J01CA01	6000	29	580.0	3480.0	4.24	25.44	4.24-580.0	136.8	13579.2
3.	Cefazolin	1000	Inj.	J01DB04	3000	11	158.0	474.0	25.4	76.3	25.4-158.0	6.2	522.1
4.	Ceftriaxone	1000	Inj.	J01DD04	2000	49	98.0	196	51.0	102	51.0-98.0	1.9	92.2
5.	Cephalexin	500	Tab.	J01DB01	2000	10	19.1	76.28	9.9	39.6	9.9-19.1	1.9	92.6
6.	Clindamycin	300	Tab.	J01FF01	1200	11	26.0	104	10.2	40.8	10.2-26.0	2.6	154.9
7.	Azithromycin	500	Tab.	J01FA10	300	92	66.0	66	5.7	5.7	5.7-66.0	11.6	1057.9
8.	Clarithromycin	500	Tab.	J01FA09	500	10	68.8	68.8	26.2	26.2	26.2-68.8	2.6	162.6
9.	Clindamycin	600	Inj.	J01FF01	1800	02	178.7	536.1	154.1	462.2	154.1-178.7	1.2	16
Drugs for antibiotic prophylaxis (Children)													
1.	Amoxicillin	125 mg/5 ml	Syrup	J01CA04	1500	14	152.5	152.5	11.4	11.4	11.4-152.5	13.4	1237.7
2.	Ampicillin	500	Inj.	J01CA01	6000	03	334.8	334.8	50.9	50.9	50.9-334.8	6.6	557.8
3.	Ceftriaxone	500	Inj.	J01DD04	1000	38	60.0	240.0	33.6	134.3	33.6-60.0	1.8	78.7
4.	Cephalexin	125 mg/5 ml	Syrup	J01DB01	2000	04	36.0	36.0	28.0	28.0	28.0-36.0	1.3	28.6
5.	Clindamycin	150 mg/ml	Syrup	J01FF01	1200	01	120.0	120.0					
6.	Azithromycin	100 mg/5 ml	Syrup	J01FA10	300	04	139.4	139.4	126.0	126.0	126.0-139.4	1.2	106
7.	Clarithromycin	500 mg/ml	Inj.	J01FA09	1000	03	2298.0	2298.0	2058.0	2058.0	2058-2298.0	1.2	11.7
8.	Azithromycin syrup	100 mg/ml	Syrup	J01DB01	300	35	156.0	156.0	30.0	30.0	30.0-156.0	5.2	420

**Table 2:** Cost ratio and cost variations of drugs included under the three categories recommended for dental pain and swelling ATC, Anatomic Therapeutic Chemical classification; DDD, defined daily dose; INR, Indian National Rupee.

S. No.	Items	Strength (mg)	Formulation	ATC code	DDD (mg)	No. of brands	Highest price (INR)	Maximum cost/DDD (INR/mg)	Lowest price (INR)	Minimum cost/DDD (INR/mg)	Price range (INR)	Cost ratio	% Cost variation
1.	Amoxicillin	500	Tab.	J01CA04	1500	33	14.4	43.2	2.9	8.7	2.9-14.4	2.9	189.7
2.	Cephalexin	500	Tab.	J01DB01	2000	10	19.1	76.28	9.9	39.6	9.9-19.1	1.9	92.6
3.	Azithromycin	500	Tab.	JO1FA10	300	92	66.0	66	5.7	5.7	5.7-66.0	11.6	1057.9
4.	Azithromycin	250	Tab.	JO1FA10	300	74	34.9	34.9	6.8	6.8	34.9-6.8	5.1	413.3
5.	Clindamycin	600	Tab.	J01FF01	1200	11	26.0	104	10.2	40.8	10.2-26.0	2.6	154.9
6.	Metronidazole	400	Tab.	J01XD01	1500	09	14.9	14.9	2.4	0.6	2.4-14.9	24.8	2383.3
7.	Amoxicillin + clavulanic acid	500 + 125	Tab.	J01CR02	1500	104	38.0	114.0	10.6	31.8	10.6-38.0	3.6	258.5

Under the affordability analysis, when the most expensive brands of drugs were analyzed, the course comprising of tablet amoxicillin + clavulanic acid (500 + 125 mg) prescribed thrice a day for seven days was the most expensive regimen (Rs. 798.0). The lowest-paid unskilled worker had to lose 2.1 days of wages to afford this regimen. When the least expensive brands of these drugs were analyzed, the most affordable medication was tablet amoxicillin 500 mg prescribed thrice a day for seven days (Rs. 60.9). The lowest-paid unskilled worker had to lose 0.2 days of wages to afford this regimen.

Only one regimen comprising of tablet cephalexin 500 mg four times a day for seven days was recommended under category B. The total cost of this regimen was Rs. 534.8 for the most expensive brand, and the lowest unskilled worker had to lose 1.4 days of wages to afford this treatment. The total cost was Rs. 277.2 for the least expensive brand corresponding to 0.7 days of loss of wages.

Under category C, four drugs were recommended under the first line of treatment, and three drugs were included under the second line of treatment. When the most expensive brands were considered, the highest cost was for taking a seven-day course of tablet clindamycin 300 mg four times a day (Rs. 728.0). This cost corresponded to 1.9 days loss of wages for the lowest-paid unskilled worker. When the least expensive brands were analyzed, the most affordable course was tablet azithromycin 500 mg for one day followed by tablet azithromycin 250 mg for four days (Rs. 32.9). This cost corresponded to a 0.1-day loss of wages (Tables 3, 4, 5).

**Table 3:** Affordability analysis of drug regimens prescribed for pain and swelling for patients not allergic to penicillin (Category A) INR, Indian National Rupee.

S. No.	Drug	Strength	Schedule	Length of treatment (days)	Total no. of units	Unit price (lowest) (INR)	Total cost (INR)	Total cost of regimen (INR)	Affordability (loss of wages in days)	Unit price (highest) (INR)	Total cost (INR)	Total cost of regimen (INR)	Affordability (loss of wages in days)
First regimen													
1.	Tab. Amoxicillin	500 mg	8 hourly	7	21	2.9	60.9	60.9	0.2	14.4	302.4	302.4	0.8
Second regimen													
1.	Tab. Amoxicillin	500 mg	8 hourly	7	21	2.9	60.9	73.5	0.2	14.4	302.4	615.3	1.6
2.	Tab. Metronidazole	500 mg	8 hourly	7	21	0.6	12.6			14.9	312.9		
Third regimen													
1.	Tab. Amoxicillin + clavulanic acid	500 + 125 mg	8 hourly	7	21	10.6	222.6	222.6	0.6	38.0	798.0	798.0	2.1

**Table 4:** Affordability analysis of drug regimens prescribed for pain and swelling for patients allergic to penicillin and for those who do not have a history of anaphylaxis, angioedema, or hives with penicillin, ampicillin, or amoxicillin (Category B) INR, Indian National Rupee.

S. No.	Drug	Strength	Schedule	Length of treatment (days)	Total no. of units	Unit price (lowest) (INR)	Total cost (INR)	Total cost of regimen (INR)	Affordability (loss of wages in days)	Unit price (highest) (INR)	Total cost (INR)	Total cost of regimen (INR)	Affordability (loss of wages in days)
First regimen													
1.	Tab. Cephalexin	500 mg	6 hourly	7	28	9.9	277.2	277.2	0.7	19.1	534.8	534.8	1.4

**Table 5:** Affordability analysis of drug regimens prescribed for pain and swelling for patients allergic to penicillin and who have a history of anaphylaxis, angioedema, or hives with penicillin, ampicillin, or amoxicillin (Category C) INR, Indian National Rupee.

S. No.	Drug	Strength	Schedule	Length of treatment (days)	Total no. of units	Unit price (lowest) (INR)	Total cost (INR)	Total cost of regimen (INR)	Affordability (loss of wages in days)	Unit price (highest) (INR)	Total cost (INR)	Total cost of regimen (INR)	Affordability (loss of wages in days)
First line of drugs													
1.	Tab. Azithromycin	500 mg	24 hourly	1	1	5.7	5.7	32.9	0.1	66.0	66.0	205.6	0.5
2.	Tab. Azithromycin	250 mg	24 hourly	4	4	6.8	27.2			34.9	139.6		
Or													
1.	Tab. Clindamycin	300 mg	6 hourly	7	28	10.2	285.6	285.6	0.8	26.0	728.0	728.0	1.9
Second line of drugs													
1.	Tab. Cephalexin	500 mg	6 hourly	7	28	9.9	277.2	289.8	0.8	19.1	534.8	534.8	1.4
2.	Tab. Metronidazole	500 mg	8 hourly	7	21	0.6	12.6			14.9	312.9		
Or													
1.	Tab. Azithromycin	500 mg	24 hourly	1	1	5.7	5.7	45.5	0.1	66.0	66.0	518.5	1.4
2.	Tab. Azithromycin	250 mg	24 hourly	4	4	6.8	27.2			34.9	139.6		
3.	Tab. Metronidazole	500 mg	8 hourly	7	21	0.6	12.6			14.9	312.9		
Or													
1.	Tab. Clindamycin	300 mg	6 hourly	7	28	10.2	285.6	285.6	0.8	26.0	728.0	728.0	1.9
2.	Tab. Metronidazole	500 mg	8 hourly	7	21	0.6	12.6			14.9	312.9		

## Discussion

In 2019, the World Health Assembly expressed its serious concerns about the high costs of medicines and medical products, which hinder the progress toward achieving universal health [10]. The cost of medicines has to be periodically assessed to determine the affordability of patients. However, such pharmacoeconomic studies on the use of medicines for dental conditions are limited. In this article, we found a wide cost variation between the various brands of antibiotics recommended by the ADA for dental pain, swellings, and post-procedural pain management. Among the penicillins, the cost ratio and cost variation for tablet amoxicillin + clavulanic acid (500 + 125 mg) was 3.6% and 258.5%, respectively, and these values were similar to a previous study by Meena et al. in 2021 [7]. The cost ratios for tablet clindamycin 600 mg and tablet cephalexin 500 mg were 2.6 and 1.9, respectively. These values were similar to a previous study with a cost ratio of 1.81 and 1.90 for tablet clindamycin and tablet cephalexin, respectively [11].

Out of all the drugs included for analysis, injection ampicillin 500 mg had the highest cost variation of 13579.2%. Ampicillin is a salient drug that serves against a broad spectrum of infections including conditions like listeriosis with high mortality rates [12]. Such a huge variation in price between the different brands could be attributed to the invaluable nature of the drug ampicillin, which is widely used for bacterial infections in monotherapy or combination therapy. Among the antibiotics, the minimum cost variation observed was 11.7% for injection clarithromycin. This was similar to a previous study where the least cost variation among all the antibiotics was 9% [13]. The cost variation among the brands of tablet azithromycin 250 mg and tablet amoxicillin 500 mg was 413.3% and 189.7%, respectively. These values were higher than the values obtained in an earlier study with a cost variation of 344% for azithromycin and 79% for amoxicillin [13]. The difference in cost variation between our study and the previous study may be because the earlier study was carried out in 2016 and there has been a definite increase in the number of brands for azithromycin and amoxicillin in the past five years.

Out of all the oral formulations (tablet form) included in this analysis, the maximum price was more than twice the minimum price available in the market. Our results were similar to Panth et al. who observed the price variations among oral antibiotics available in Nepal [14]. Among the parenteral drugs included in our study, the cost ratio of ceftriaxone 500 mg was 1.8 among a total of 38 brands. This was similar to a previous study by Mir where they had analyzed 37 brands of ceftriaxone 500 mg and observed a cost ratio of 1.8 [13]. Among the analgesics, tablet acetaminophen 500 mg was more expensive and had a higher cost variation. The most expensive brand of acetaminophen was 2.1 times higher than the most expensive brand of tablet ibuprofen. Ibuprofen was more affordable as the most expensive brand costs Rs.1.6 per unit, which was similar to a previous study with a cost of Rs. 2.95 per unit [15].

In the present analysis, when the most expensive brands of the regimens recommended for pain and swelling were analyzed, only one regimen in category A and one regimen in category C required less than one-day wages to purchase them. All the other drugs recommended under the other categories required loss of wages of more than one day. The most expensive regimen was tablet amoxicillin and clavulanic acid (500 + 125 mg) three times a day for seven days costing 2.1 days of loss of wages. When the most expensive brands were analyzed, 77.8% of the drug regimens were unaffordable and only 22.2% were affordable. When the least expensive brands were analyzed, all the drugs recommended under the three categories were affordable and required a loss of wages of less than one day. The average loss of wages was 0.5 days in the least expensive group (Tables 3-5).

## Conclusions

This article compared the various brands of antibiotics and analgesics available in the Indian market and observed wide cost variations in the drugs routinely used in dentistry. With such vast numbers of brands available in the market, it is fundamental that dental surgeons select drugs carefully considering their quality, availability, and costs. Increasing the availability of medicines in public health sectors and prescription of generic medications can help reduce the economic burden of the public and promise a better prognosis. One of the key objectives of the National Oral Health Policy of India 2021 is to provide affordable and safe medicines along with quality dental care to the population. Training in pharmacoeconomics should be introduced in dental schools to guide young dentists about the pricing policies and affordability issues.
